# Supplementary material for: Limited Response of Indigenous Microbes to Water and Nutrient Pulses in High-Elevation Atacama Soils: Implications for the Cold–Dry Limits of Life on Earth
Source: Microorganisms. 2020 Jul 16;8(7):1061. doi: 10.3390/microorganisms8071061 (PMC7409055; doi:10.3390/microorganisms8071061)
Supplement: Supplementary file 1 [file microorganisms-08-01061-s001.pdf]

Supplementary Material

Limited response of indigenous microbes to water and nutrient pulses in high-elevation Atacama soils: implications for the cold-dry limits of life on Earth

Lara Vimercati[1], Clifton P. Bueno de Mesquita[1,2] and Steven K. Schmidt[1,*]

[1]Department of Ecology and Evolutionary Biology, University of Colorado, Boulder, CO, USA 80309-0334

[2]Institute of Arctic and Alpine Research, University of Colorado, Boulder, CO, USA 80309-0450 *Corresponding author Steve.Schmidt@colorado.edu

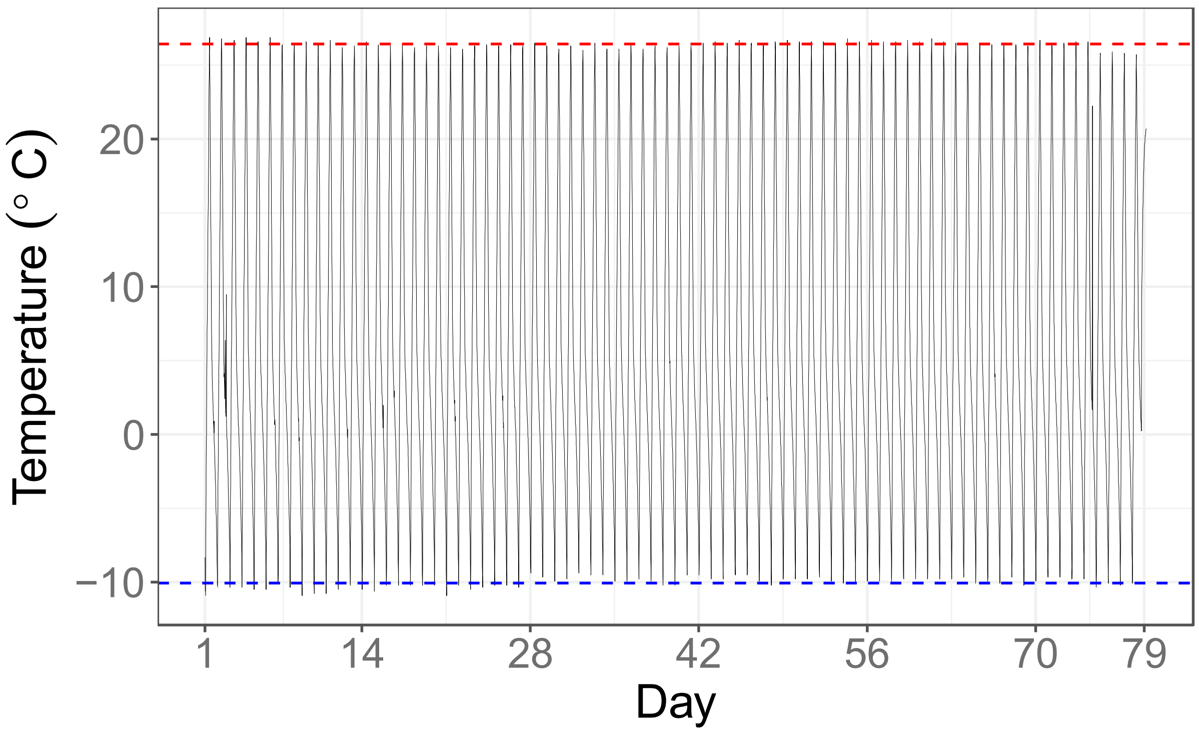

Supplementary Figure 1. Growth chamber temperature data from a HOBO data logger throughout the course of the experiment. The red dashed line shows the average daily maximum of 26.42˚C while the blue dashed line shows the average daily minimum of -10.07˚C.

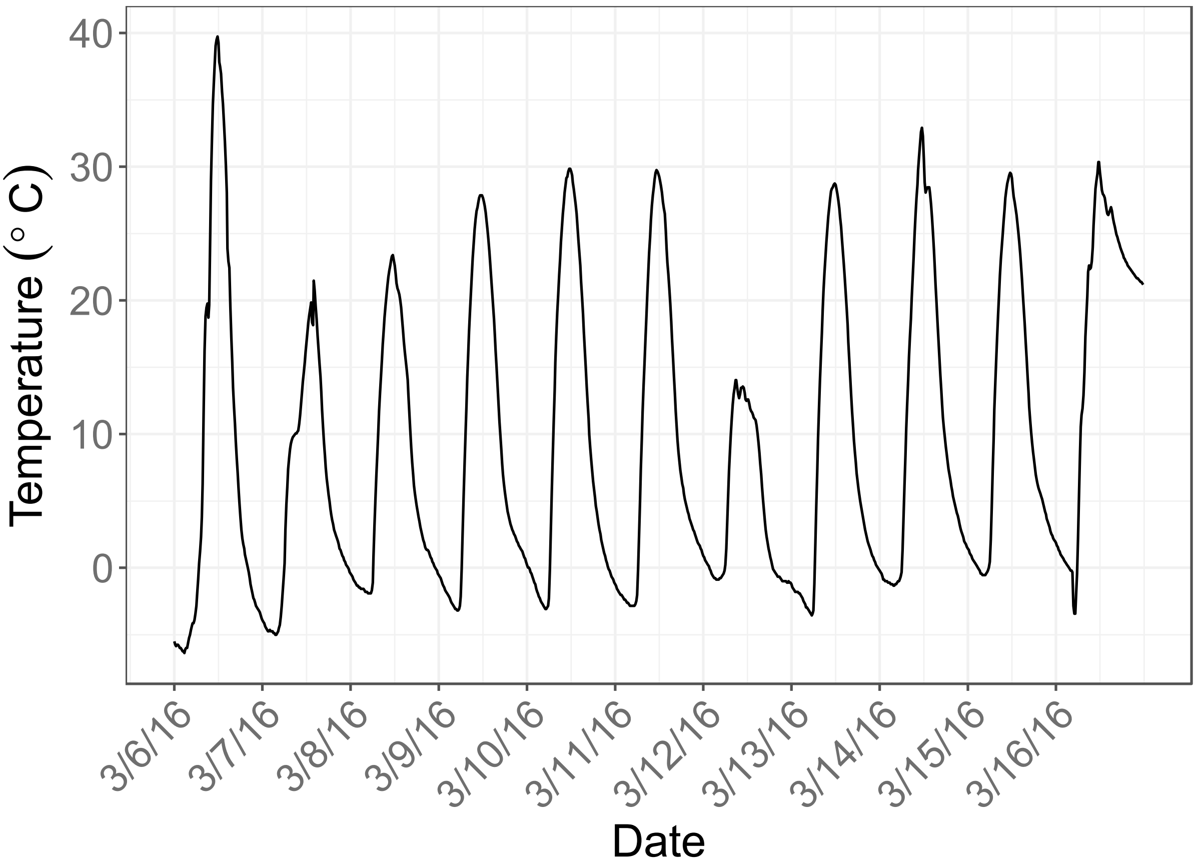

Supplementary Figure 2. Eleven days of soil temperature data at 4 cm depth from a HOBO data logger deployed at the site of the field experiment.

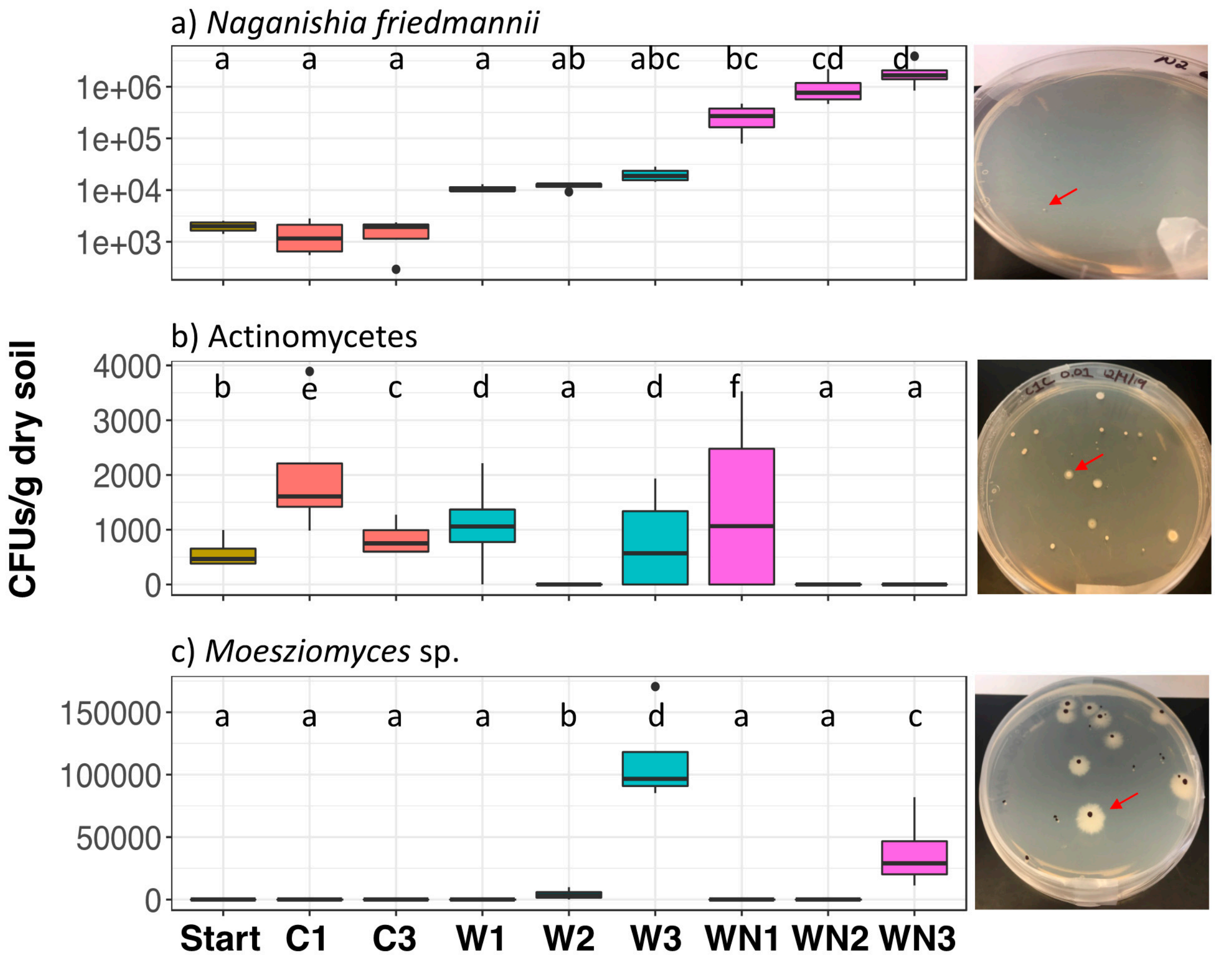

Supplementary Figure 3. Number of colony forming units per gram of dry soil among all treatments in the microcosm experiment for a) *Naganishia friedmannii*, b) Actinomycetes and c) *Moesziomyces* sp. Note the difference in the y-axis scale among the three panels. Different letters represent significant pairwise differences between treatments (Tukey HSD, $p < 0.05$). Also shown are photos of the colonies on the plates. *N. friedmannii* appears as small, opaque, smooth, round, slowly growing colonies that become visible after ~1 week. Viewed under the microscope, cells appear as classic spherical yeast cells approximately 5 µm in diameter. Since we have also viewed these cells in pure culture and confirmed their identity with DNA sequencing in a previous experiment [3], we can say with high confidence that these are *N. friedmannii* colonies. Actinomycetes appear as slightly larger colonies, featuring a darker, raised center area followed by a flat, lighter-colored outer ring. Viewed under the microscope, cells from these colonies were thin and hair-like in shape, approximately 1 µm in size. *Moesziomyces* sp. appears as large, fuzzy, white colonies. Viewed under the microscope, cells are dimorphic, featuring both ovoid yeast-type cells as well as hyphae. *Moesziomyces* sp. are reported to be dimorphic [72] and sequencing data showed they increased with multiple water additions; both of these pieces of information suggest that these colonies are likely *Moesziomyces* sp. Note that the photo of the *N. friedmannii* plate is from a field sample and was taken just after the first colony was visible and thus does not show many colonies.

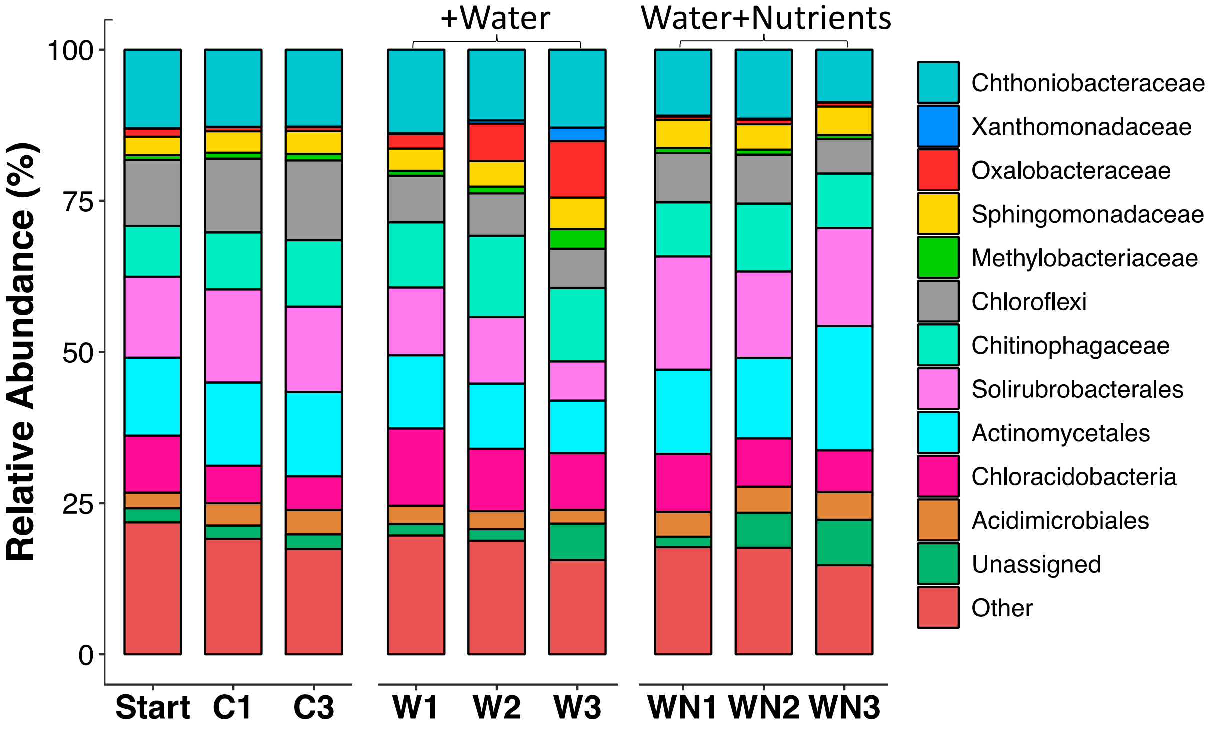

Supplementary Figure 4. Taxonomy of bacterial community shift in response to +W and +WN amendments in the microcosm experiment**.** Stacked bar graphs showing relative abundances of dominant eukaryotic taxa (from 18S sequencing data). Taxonomy is from the Greengenes database. Bars represent means (n=4). Most of the “Other” category in the starting soil is plant material [3].
